# Prognostic impact of syndecan-1 expression in invasive ductal breast carcinomas

**DOI:** 10.1038/sj.bjc.6604400

**Published:** 2008-06-10

**Authors:** D Loussouarn, L Campion, C Sagan, J-S Frenel, F Dravet, J-M Classe, R Pioud-Martigny, D Berton-Rigaud, E Bourbouloux, J-F Mosnier, F-R Bataille, M Campone

**Affiliations:** 1 Department of Pathology, University Hospital of Nantes, Nantes, France; 2 Department of Statistics, Centre René Gauducheau, CLCC Nantes-Atlantique, Saint-Herblain/Nantes cedex, Saint-Herblain, France; 3 Department of Medical Oncology, Centre René Gauducheau, CLCC Nantes-Atlantique, Saint-Herblain/Nantes cedex, Saint-Herblain, France; 4 Department of Surgery, Centre René Gauducheau, CLCC Nantes-Atlantique, Saint-Herblain/Nantes cedex, Saint-Herblain, France; 5 Department of Biology, Centre René Gauducheau, CLCC Nantes-Atlantique, Saint-Herblain/Nantes cedex, Saint-Herblain, France

**Keywords:** syndecan-1, invasive ductal carcinoma, epithelial–mesenchymal transition, stroma, prognostic factor

## Abstract

Carcinoma cells lack syndecan-1 expression when they are transiting from an epithelial to a less-differentiated mesenchymal phenotype (epithelial–mesenchymal transition, EMT). Furthermore, a shift of syndecan-1 expression from malignant epithelial cells to reactive stromal cells has also been observed during progression of many carcinomas. Finally, epithelial and/or stromal syndecan-1 expression is of prognostic value in many carcinomas. Because recent results are contradictory in breast carcinomas, we have re-evaluated the prognostic significance of syndecan-1 expression in a cohort of 80 patients with invasive ductal breast carcinomas. The tumours from 80 patients diagnosed with invasive ductal breast carcinomas were used to construct a tissue microarray, which was stained with syndecan-1 by immunohistochemistry. We correlated syndecan-1 expression with clinicopathologic parameters and relapse-free survival (RFS). Exclusive epithelial expression of syndecan-1 is observed in 61.25% of the patients, whereas exclusive stromal expression is observed in 30% of the patients. Only 8.75% of the patients had both stromal and epithelial expressions of syndecan-1. A significant correlation was found between the loss of syndecan-1 epithelial expression and the syndecan-1 stromal expression with high grade of malignancy (*P*=0.011). The loss of syndecan-1 epithelial expression is correlated with RFS (*P*=0.001). Using multivariate Cox analysis, loss of epithelial syndecan-1 expression was the only prognostic indicator (*P*<0.001). We concluded that the loss of syndecan-1 epithelial expression was of strong prognostic value in breast carcinomas.

Syndecan-1, a transmembrane heparan sulphate proteoglycan, is expressed by many normal cells, including epithelial and endothelial cells. It is also expressed in carcinoma cells. Syndecan-1 is an antigen of differentiation that is lost by carcinoma cells when these malignant cells are transiting from an epithelial to a less-differentiated mesenchymal phenotype (epithelial–mesenchymal tumoral transition, EMT) (Kato *et al*, 1995; Leppä *et al*, 1996 and reviewed in Larue and Bellacosa, 1996). Of major interest, a shift of syndecan-1 expression from malignant epithelial cells to reactive stromal cells has also been observed in many carcinomas during their progression (Mennerich *et al*, 2004). Epithelial and/or stromal syndecan-1 expression is of prognostic value in many carcinomas, but because recent results are contradictory in breast carcinomas (Barbareschi *et al*, 2003; Leivonen *et al*, 2004), we have re-evaluated the prognostic significance of syndecan-1 expression in a homogeneous cohort of patients with invasive ductal breast carcinomas.

## PATIENTS AND METHODS

### Patients

We included 80 patients with breast cancer, considered as good prognosis, between January 1995 and December 2001 at the René Gauducheau Comprehensive Cancer Center (Nantes, France), according to the following criteria: no chemotherapy in adjuvant and neoadjuvant setting, histological diagnosis of invasive ductal carcinoma, no pathological lymph node invasion, no distant metastasis, no other previous or concomitant primary malignancies. The mean age was 72.7 years (44–95 years). The main clinicopathological features are listed in Table 1. Patients were staged according to the International Union against Cancer Node Metastasis (UICC-TNM) Classification. All patients underwent tumorectomy (*n*=65) or mastectomy (*n*=15) with auxillary lymph node dissection. Seventy-four patients (92.5%) received post-operative irradiation and 66 (82.5%) hormone therapy. The patients who did not receive hormone therapy were those presenting with a contre-indication. None of these patients received chemotherapy in adjuvant or neoadjuvant setting. The median follow-up among survivors was 7.1 years (range, 4.5–9.9 years).

### Tissue samples and tissue microarray construction

Tissue specimen was fixed in 10% buffered formalin and routinely processed. Tumour grading was performed according to Scarff–Bloom–Richardson grading modified by Elston and Ellis (1991).

The arrays were constructed with the 1 mm punch of the Beecher arrayer. Haemalun–eosin–safran-stained sections of breast ductal invasive carcinoma were reviewed and the area of interest marked out on the slide. This area of interest corresponded to both stroma and carcinoma cells. Three cores with a diameter of 1 mm were punched out from the paraffin-embedded tissue block in each case and incorporated into a final tissue array block. A control tissue sample (normal breast) was included in the final array block.

Before immunostaining, an HES-stained section of the final array block was examined to confirm the representative areas of the tumours in comparison with the original HES-stained section.

### Immunohistochemistry

Sections (4 *μ*m) were cut from the tissue microarray blocks and placed on superfrost slides. The immunochemical technique was performed on an automated immunostainer (Lab Vision, Fremont, USA) using the streptavidin–biotin amplification technique (ChemMate kit; Dako, Glostrup, Denmark) after appropriate antigen retrieval in EDTA buffer (pH 8) at 95°C. It involved the application of the specific primary antibodies to Syndecan-1/CD138 (clone MI15, dilution 1 : 100; Dako), estrogen receptor (6F11, 1 : 100; Novocastra, Newcastle, UK), progesterone receptor (PgR636, 1 : 100; Dako), and HER2 (1 : 800; Dako). Peroxidase activity was revealed using 3,3′-diaminobenzidine for 5 min. Sections were counterstained with Harris haematoxylin for 3 min. Negative controls were obtained by omitting primary antibodies. Positive internal controls were normal ducts.

### Immunohistochemistry interpretation

Immunohistochemical staining was assessed in both epithelial and stromal components. The proportion of cells stained as well as the intensity of staining was evaluated. The percentage of imunoreactive carcinoma cells was evaluated by two observers. Syndecan-1 immunostaining intensity (of the carcinoma cells and stroma) was scored as follows: 0, no staining; 1+, weak; 2+, moderate; 3+, strong. The level of epithelial immunostaining was graded by scoring the percentage of syndecan-1-positive carcinoma cells into two groups as negative (<10% of the cancer cells positive) and positive (>10% carcinoma cells positive).

The level of stromal immunostaining was graded by scoring the percentage of positivity into two groups: negative (<10%) and positive (>10%).

### Statistical analysis

Categorical data, presented as frequencies, were compared using the Fisher exact test and continuous variables, expressed as median (range) was compared using the Mann–Whitney or the Kruskal–Wallis test as appropriate. Event for disease-free survival was relapse (local or distant). Relapse-free survival (RFS) curves were estimated using the Kaplan–Meier method and compared at univariate step by the means of log-rank tests. Cox proportional hazards regression analyses were performed on epithelial expression (discretized and continuous) and on all other known prognostic parameters to assess the independent association of them to RFS. Because of the small number of relapses during follow-up (*n*=13/80), we were concerned about the possibility of model overfitting. To confirm the results obtained on the original data set, we decided to use permutation test at the univariate step and bootstrapping techniques at the multivariate step. In univariate analysis, we determined for each parameter from 10 000 new data sets derived from random permutation sampling the probability that new *P*-value was greater than that from the original data set. In multivariate analysis, we created 10 000 new data sets derived from random bootstrap sampling and replacement of the main cohort set, and we performed a stepwise Cox regression analysis on each data set. We counted the number of models each of the parameters entered (10 000 possible) and estimated the distribution of *β*-coefficients (median, range) if a parameter was present in more than a half of simulations. In order to obtain very extreme values, range was expressed from 1st percentile to 99th percentile. All data were analysed with SAS version 9.1 (SAS Institute, Cary, NC, USA) and STATA 8.2 SE (StataCorp, College Station, TX, USA).

## RESULTS

### Syndecan-1 expression

Intense staining on the basolateral surface of epithelial cells from normal ducts demonstrates characteristic localisation of syndecan-1. No immunostaining was observed in normal stromal tissue.

**Figure 1:**
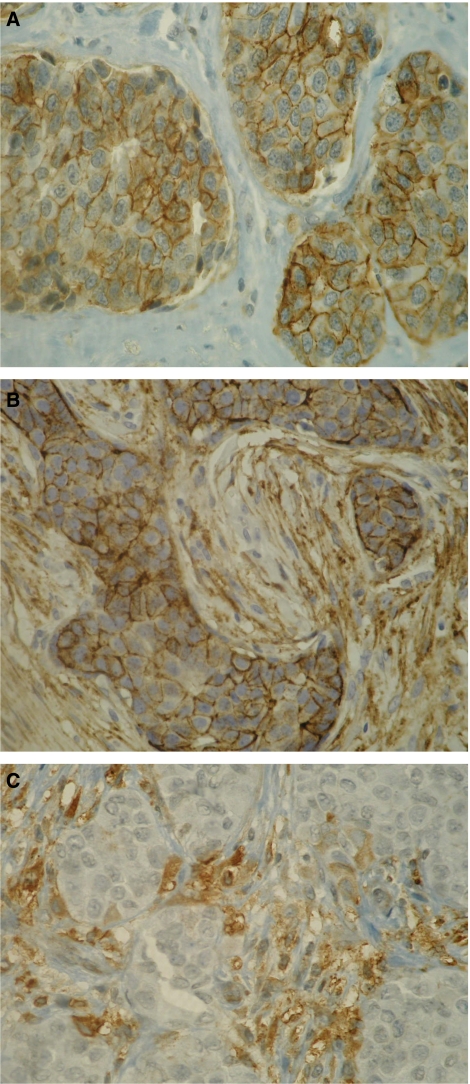
(**A**) Intense membranous immunostaining of carcinoma cells; no stromal immunostaining (E+/S−). (**B**) Immunostaining of both carcinoma cells and stroma (E+S+). (**C**) Stromal immunostaining and no epithelial immunostaining (E−S+).

Syndecan-1 expression in breast carcinoma specimen was observed in epithelial tumour cells, stromal component, or both. Both epithelial and stromal components showed a very homogeneous staining. Only epithelial syndecan-1 immunostaining was observed in 49 out of 80 patients (61.25%) (Figure 1A). The median percentage of positive carcinoma cells was 100% (range: 30–100%). The epithelial immunostaining was associated with stromal expression in 7 out of 80 patients (8.75%) (Figure 1B). In these cases, the median percentage of positive carcinoma cells was 100% (range: 50–100%). Only stromal syndecan-1 expression was observed in 24 out of 80 patients (30%) (Figure 1C). Immunostaining was seen both in stromal cells and in collagen tissue. The median percentage of stroma immunoreactivity was 100% (range: 70–100%). In all cases, intensity of epithelial and stromal immunostaining was strong (3+).

### Syndecan-1 expression correlates

Baseline characteristics of the patients according to their epithelial expression status were described in Table 1. A high syndecan-1 expression in epithelial tumour cells was strongly associated with low histological grade (*P*<0.011). Conversely, the absence of epithelial expression correlated with the highest histological grade. Generally, higher levels of syndecan-1 immunostaining were observed in the well-differentiated tumours, whereas significant reduction of syndecan-1 immunostaining was observed in poorly differentiated tumours.

### Baseline characteristics and univariate RFS analysis

Median follow-up among survivors was 7.1 years (range, 4.5–9.9 years). There were 13 relapses; 9 metastasis; and 4 local relapses.

**Figure 2:**
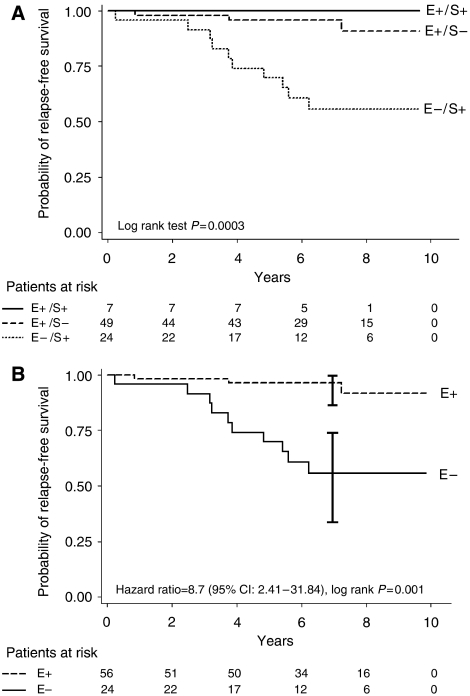
Kaplan–Meier RFS curves according to epithelial status. (**A**) Separate epithelium/stroma status. (**B**) Epithelium positive *vs* epithelium negative status.

At median follow-up (7 years), RFS was not different between patients with exclusive epithelial expression (E+S−) and patients with epithelial and stromal expression (E+/S+) (95.6 *vs* 100%, *P*=0.54). Patients with exclusive stromal expression (E−S+) had 7-year RFS significantly lower than patients with exclusive epithelial expression (E+S−) (55.6 *vs* 95.6%; *P*<0.001) or associated with stromal expression (E+S+) (36 *vs* 100%, *P*=0.055). So, E+S+ and E+S− groups were pooled and analysed compared with the E−S+ group for further analyses (Figure 2A).

A strong association (hazard ratio (HR)=8.7/*P*=0.001) was found between the loss of epithelial expression and RFS. Patients with epithelial expression (exclusive or associated with stromal expression) had excellent outcome (7-year Kaplan–Meier RFS: 96%; 95% CI: 86–99%), whereas patients without epithelial expression had worse outcome (7-year Kaplan–Meier RFS: 56%; 95% CI: 33–73%) (Figure 2B). No significant association was found in our population between RFS and the other known prognostic factors (age, Elston and Ellis grade, histologic tumour size, pT stage, hormone therapy, and PR or ER status). The result of the loss of epithelial expression was reinforced by permutation test (detailed tests results in Table 2).

### Multivariate RFS analysis

To confirm accuracy of group repartition (E+S+ and E+S- *vs* E−S+), first Cox analysis on original data set was made only on the two opposite groups (E+S− *vs* E−S+). Comparison of this restricted model with the other two complete models (model 2: E+S− and E+S+ *vs* E−S+/model 3: E+S− *vs* E+S+ and E−S+) confirmed better fit of model 2 (E+ *vs* E−) with restricted one than model 3 (S+ *vs* S−) (data not shown).

On original data set, epithelial expression loss HR was 10.92 (95% CI: 2.8–42.6), with *P*<0.001 if discretized as E− *vs* E+ and was 13.69 (95% CI: 3.03–61.80), with *P*<0.001 if continuous. No other parameter was significantly associated with RFS (Table 3).

**Figure 3:**
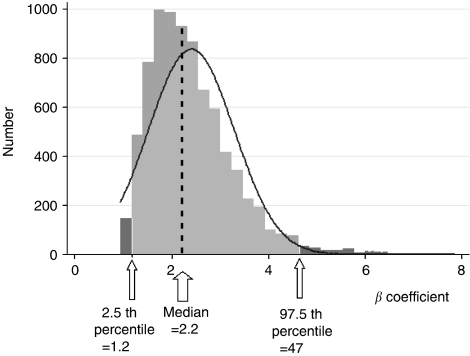
Estimate distribution of absence of epithelial expression (10 000 bootstraped data sets).

The results of 10 000 bootstrap-based stepwise Cox proportional hazards analyses strengthened the independent role of epithelial expression in RFS and confirmed its HR level: this parameter entered 8511 models (10 000 possible) with a estimate *β*-median value of 2.25 (95% CI: 1.21–4.67) (corresponding to HR=9.52; 95% CI: 3.3–106.3) with a *P*-value median of 0.0015 (95% CI: 1 10-4 to 0.0304) (Figure 3).

## DISCUSSION

The progression of epithelial tumours is characterised by (i) a loss of syndecan-1 expression in tumour cells (EMT) and (ii) a shift of syndecan-1 expression from tumour cells to stroma. First, EMT is the conversion of malignant epithelial cells into cells with a mesenchymal phenotype (Larue and Bellacosa, 2005). A critical feature of EMT is the downregulation of both E-cadherin and syndecan-1 expression on tumour cells (Kato *et al*, 1995; Leppä *et al*, 1996). Second, the expression of syndecan-1 in the stroma is characterised by the appearance of a strong immunoreactivity for syndecan-1 in the reactive stroma of invasive carcinomas (Mennerich *et al*, 2004). Such stroma immunoreactivity, that corresponds to a true induction of syndecan-1 synthesis by reactive stromal cells and not simply to the fixation of shedded syndecan-1 to stromal cells (Mennerich *et al*, 2004), has been initially described in invasive breast carcinomas by Stanley *et al*, (1999) and confirmed by others (Wiksten *et al*, 2001; Mennerich *et al*, 2004). Epithelial–mesenchymal transition is associated with a more aggressive Akt/PI-3K signalling pathway within carcinoma cells and with clinically more aggressive tumours (Larue and Bellacosa, 2005). Furthermore, experimental and clinical data have shown that the expression of syndecan-1 in the stroma promotes breast carcinoma growth *in vivo* and stimulates tumour angiogenesis (Maeda *et al*, 2004, 2006). In summary, the loss of syndecan-1 by carcinoma cells leads to the appearance of a more aggressive phenotype and behaviour of carcinoma cells, whereas the expression of syndecan-1 in the reactive stromal cells creates a favourable microenvironment for tumour cell growth and angiogenesis.

In breast carcinomas, three studies have been devoted to the expression of syndecan-1 (Stanley *et al*, 1999; Barbareschi *et al*, 2003; Leivonen *et al*, 2004). Stanley *et al* (1999) were the first to describe the induction of syndecan-1 expression in the stroma of invasive breast carcinomas, but the small number of patients (*n*=20) did not permit any statistical study. The two other studies are contradictory. Barbareschi *et al* (2003) have shown that syndecan-1 is expressed at high levels in a significant percentage of breast carcinomas and that this high expression is related to a poor clinical behaviour. Stromal syndecan-1 expression was not considered on a prognostic point of view. Leivonen *et al* (2004) have also shown the poorer prognosis of breast carcinoma patients expressing syndecan-1 within their tumour cells, but the better prognosis of those lacking syndecan-1 expression within the stroma. Both studies were in disagreement with many other studies showing that the loss of syndecan-1 expression within carcinoma cells, rather than its high expression, was of poor prognostic value, as well as expression of syndecan-1 in the stroma (Mennerich *et al*, 2004; Larue and Bellacosa, 2005, and more specifically Inki *et al*, 1994; Matsumoto *et al*, 1997; Nackaerts *et al*, 1997; Anttonen *et al*, 1999; Conejo *et al*, 2000; Juuti *et al*, 2005). Finally, both studies suggested that breast carcinomas could be an exception within carcinomas.

For these reasons, we have re-evaluated the prognostic value of syndecan-1 in patients with invasive breast carcinomas. The interest of our cohort of patients is its homogeneity: one histological type of breast carcinoma, homogeneity of treatment, especially lack of chemotherapy, in contrast to the other studies devoted to breast cancers. Our study shows that 61.25% of our patients with invasive ductal breast carcinomas overexpressed syndecan-1 within their carcinoma cells, whereas lacking it in the stroma (E+S−) and that 30% lacked syndecan-1 within the tumour cells but expressed it in their stroma (E−S+). Only 8.75% of the patients retained syndecan-1 expression within their tumour cells while having reactive stroma-expressing syndecan-1 (E+S+). This last group of patients is too small to allow any further investigation and to draw any conclusion. Our correlates and survival studies have shown that these two processes correlated with high grade of malignancy and with poor RFS. In all the cases, however, correlations were statistically more significant with the loss of syndecan-1 epithelial expression. Finally, our multivariated analyses have shown that the loss of syndecan-1 epithelial expression was the strongest prognostic predictor of survival in these patients. Our data are in agreement with other studies showing that both loss of syndecan-1 epithelial expression and syndecan-1 stromal expression are associated with poor clinical outcome in many cancers and in addition show that this is also true in cases of invasive ductal breast carcinomas. Further investigations will be necessary to (i) evaluate more patients with different histological types of breast carcinomas (and different types of treatments); (ii) define the gene profiling of these new different types of patients (E+S−, E-S+, and the rare E+S+) in comparison with previously published gene profiling of ductal carcinomas (Sorlie *et al*, 2001); and (iii) understand the simultaneous or dissociated mechanisms in charge of these processes.

## Figures and Tables

**Table 1:** Baseline characteristics of 80 patients according to syndecan-1 epithelial and/or stromal expression

**Variable**	**E+ (S+ou−) *n*=56**	**E− (S+) *n*=24**	***P*-value**
*Age (years)*			
Median	73	76	
Range	(44–95)	(50–91)	0.194
			
*Histologic size (mm)*			
Median	20	21	
Range	(10–40)	(7–35)	0.458
			
*pT*			
pT1	32	12	
pT2	24	12	0.628
			
*EE grade*			
I	20	2	
II	30	15	
III	6	7	0.011
			
*ER*			
+	53	24	
−	3	0	0.550
			
*PR*			
+	47	20	
−	9	4	1.000
			
*Hormone therapy*			
No	10	4	
Yes	46	20	1.000
			
*HER2*			
0, 1, 2 +	54	23	
3+	2	1	1.000

E=epithelial staining (E+=56 patients, E−=24 patients); EE grade=Elston and Ellis grade; ER=oestrogen receptor; PR=progesterone receptor; S=stromal staining.

**Table 2:** Univariate relapse free log-rank analysis

**Variable**	**HR (95% CI)**	***P*-value (original set)**	***P*-value permutation (*n*=10 000)**
Epithelial expression (negative *vs* positive)	8.76 (2.41–31.83)	0.001	0.0001
Epithelial expression (continuous)	10.79 (2.50–46.56)	0.001	0.0003
pT stage (pT2 *vs* pT1)	0.55 (0.17–1.80)	0.324	0.377
Age (years)	0.97 (0.93–1.02)	0.330	0.310
			
*SBR grade*			
II *vs* I	1.07 (0.28–4.14)	0.923	1.000
III *vs* I	1.62 (0.33–8.07)	0.573	0.586
			
Hormone therapy (yes *vs* no)	0.67 (0.18–2.47)	0.550	0.562
Her2 (3+ *vs* 0, 1, 2+)	2.74 (0.35–21.21)	0.334	0.395
PR (negative *vs* positive)	2.39 (0.73–7.79)	0.148	0.062
ER (negative *vs* positive)	—	NC^*^	NC^*^

ER=oestrogen receptor; HR=hazard ratio; PR=progesterone receptor.

^*^NC, not calculable.

**Table 3:** Multivariate Cox relapse-free analysis

**Variable**	**HR**	**HR 95% CI**	***P*-value**	**Bootstrap stepwise Cox (*n*/10 000)**
Epithelial status (E− *vs* E+)	11.43	2.94–44.43	<0.0001	8511
pT stage (pT2 *vs* pT1)	0.37	0.06–2.34	0.293	2303
Age (years)	0.96	0.91–1.02	0.227	3974
SBR (III *vs* I, II)	1.97	0.25–15.62	0.521	1573
Hormone therapy (yes *vs* no)	1.04	0.24–4.50	0.953	640
Her2 (3+ *vs* 0, 1, 2+)	2.92	0.29–29.93	0.366	1573
PR (negative *vs* positive)	2.23	0.65–7.72	0.203	3479
ER (negative *vs* positive)	NC^*^	—	—	—

ER=oestrogen receptor; HR=hazard ratio; NC=not calculable; PR=progesterone receptor.

Number of subjects=80/number of failures=13/LR *χ*^2^ (7)=21.15/Probability>*χ*^2^=0.0036.

^*^NC was dropped for non-convergence.

## References

[bib1] Anttonen A, Kajanti M, Heikkila P, Jalkanen M, Joensuu H (1999) Syndecan-1 expression has prognostic significance in head and neck carcinoma. Br J Cancer 79: 4558–4564 10.1038/sj.bjc.6690088 PMC2362450 10027330

[bib2] Barbareschi M, Maisonneuve P, Aldovini D, Cangi MG, Pecciarini L, Mauri FA, Veronese S, Caffo O, Lucenti A, Palma PD, Galligioni E, Doglioni C (2003) High syndecan-1 expression in breast carcinoma is related to an aggressive phenotype and to poorer prognosis. Cancer 98: 474–483 12879463 10.1002/cncr.11515

[bib3] Conejo JR, Kleef J, Koliopanos A, Matsuda K, Zhu ZW, Goecke H, Bicheng N, Zimmermann A, Korc M, Friess H, Büchler MW (2000) Syndecan-1 is up-regulated in pancreatic but not in other gastrointestinal cancers. Int J Cancer 88: 12–20 10962434 10.1002/1097-0215(20001001)88:1<12::aid-ijc3>3.0.co;2-t

[bib4] Elston CW, Ellis IQ (1991) Pathological prognostic factors in breast cancer I. The value of histological grade in breast cancer: experience from a large study with long-term follow-up. Histopathology 19: 403–410 1757079 10.1111/j.1365-2559.1991.tb00229.x

[bib5] Fujiya M, Watari J, Ashida T, Honda M, Tanabe H, Fujiki T, Saitoh Y, Kohgo Y (2001) Reduced expression of syndecan-1 affects metastatic potential and clinical outcome in patients with colorectal cancer. Jpn J Cancer Res 92: 1074–1081 11676858 10.1111/j.1349-7006.2001.tb01062.x PMC5926619

[bib6] Hasengaowa J, Kodama J, Kusumoto T, Shinyo Y, Seki N, Hiramatsu Y (2005) Prognostic significance of syndecan-1 expression in human endometrial cancer. Ann Oncol 1: 1109–1115 10.1093/annonc/mdi224 15851381

[bib7] Inki P, Joensuu H, Grenman R, Klemi P, Jalkanen M (1994) Association between syndecan-1 expression and clinical outcome in squamous cell carcinoma of the head and neck. Br J Cancer 70: 319–323 8054282 10.1038/bjc.1994.300 PMC2033500

[bib8] Juuti A, Nordling S, lundin J, Louhimo J, Haglund C (2005) Syndecan-1 expression. A novel prognostic marker in pancreatic cancer. Oncology 68: 97–106 15886501 10.1159/000085702

[bib9] Kato M, Saunders S, Nguyen H, Bernfield M (1995) Loss of cell surface syndecan-1 causes epithelia to transform into anchorage-independent mesenchyme-like cells. Mol Biol Cell 6: 559–576 7545031 10.1091/mbc.6.5.559 PMC301215

[bib10] Larue L, Bellacosa A (2005) Epithelial–mesenchymal transition in development and cancer: role of phosphatidylinositol 3′ kinase/AKT pathways. Oncogene 24: 7443–7454 16288291 10.1038/sj.onc.1209091

[bib11] Leivonen M, Lundin J, Nordling S, Von Boguslawski K, Haglund C (2004) Prognostic value of syndecan-1 expression in breast cancer. Oncology 67: 11–18 15459490 10.1159/000080280

[bib12] Leppä S, Vleminckx K, Van Roy F, Jalkanen M (1996) Syndecan-1 expression in mammary epithelial tumor cells is E-cadherin-dependent. J Cell Sc 109: 1393–1403 8799827 10.1242/jcs.109.6.1393

[bib13] Lundin M, Nordling S, Lundin J, Isola J, Wilsten JP, Haglund C (2005) Epithelial syndecan-1 expression is associated with stage and grade in colorectal cancer. Oncology 68: 306–313 16020957 10.1159/000086969

[bib14] Maeda T, Alexander CM, Friedl A (2004) Induction of syndecan-1 expression in stromal fibroblasts promotes proliferation of human breast cancer cells. Cancer Res 64: 612–621 14744776 10.1158/0008-5472.can-03-2439

[bib15] Maeda T, Desouky J, Friedl A (2006) Syndecan-1 expression by stromal fibroblasts promotes breast carcinoma growth *in vivo* and stimulates tumor angiogenesis. Oncogene 25: 1408–1412 16247452 10.1038/sj.onc.1209168

[bib16] Matsumoto A, Ono M, Fujimoto Y, Gallo RL, Bernfield M, Kohgo Y (1997) Reduced expression of syndecan-1 in human hepatocellular carcinoma with high metastatic potential. Int J Cancer 74: 482–491 9355969 10.1002/(sici)1097-0215(19971021)74:5<482::aid-ijc2>3.0.co;2-#

[bib17] Mennerich D, Vogel A, Klaman I, Dahl E, Lichtner RB, Rosenthal A, ohlenz HD, Thierauch KH, Sommer A (2004) Shift of syndecan-1 expression from epithelial to stromal cells during progression of solid tumours. Eur J Cancer 40: 1373–1382 15177497 10.1016/j.ejca.2004.01.038

[bib18] Nackaerts K, Verbeken E, Deneffe G, Vanderschueren B, Demedts M, David G (1997) Heparan sulfate proteoglycan expression in human lung-cancer cells. Int J Cancer 74: 335–345 9221815 10.1002/(sici)1097-0215(19970620)74:3<335::aid-ijc18>3.0.co;2-a

[bib19] Sorlie T, Perou CM, Tibshirani R, Aas T, Geisler S, Johnsen H, Hastie T, Eisen MB, Van de Rijn M, Jeffrey SS, Thorsen T, Quist H, Matese JC, Brown PO, Botstein D, Eystein Lonning P, Borrensen-Dale AL (2001) Gene expression patterns of breast carcinomas distinguish tumor subclasses with clinical implications. Proc Natl Acad Sci USA 98: 10869–10874 11553815 10.1073/pnas.191367098 PMC58566

[bib20] Stanley MJ, Stanley MW, Sanderson RD, Zera R (1999) Syndecan-1 expression is induced in the stroma of infiltrating breast carcinoma. Am J Clin Pathol 112: 377–383 10478144 10.1093/ajcp/112.3.377

[bib21] Wiksten JP, Lundin J, Nordling S, Lundin M, Kokkola A, von Boguslawski K, Haglund C (2001) Epithelial and stromal syndecan-1 expression as predictor of outcome in patients with gastric cancer. Int J Cancer 95: 1–6 11241302 10.1002/1097-0215(20010120)95:1<1::aid-ijc1000>3.0.co;2-5

